# Role of brief intervention for prenatal alcohol consumption conducted by nonmedical professionals

**DOI:** 10.1186/1940-0640-8-S1-A4

**Published:** 2013-09-04

**Authors:** Ornella Ancarani, Ilaria Londi, Paolo Borro, Valentino Patussi, Emanuele Scafato, Gianni Testino

**Affiliations:** 1 Regional Center for Alcohol, Liguria, Genova, Italy; 2 Collaborative Centre for Research and Health Promotion, World Health Organization, Rome, Italy; 3 Regional Center for Alcohol, Tuscany, Florence, Italy; 4 National Institute of Health, Rome, Italy

## Aim

We examined the efficacy of brief intervention by nonmedical professionals as a technique to help pregnant women achieve abstinence from alcohol and tested the effectiveness of a brief intervention in the reduction of prenatal alcohol consumption by women when a partner is included.

## Methods

From January 2008 to December 2011, 251 (mean age: 34+/-6) pregnant women with a T-ACE positive screen for risk of alcohol consumption while pregnant were evaluated in our regional alcohol center. The T-ACE asks four questions that give the assessment its name: Tolerance (how many drinks does it take to make you feel high?), Annoyed (have people ever annoyed you by criticizing your drinking?), Cut down (have you ever felt you ought to cut down on your drinking?), and Eye-opener (have you ever had a drink first thing in the morning to steady your nerves or get rid of a hangover?). The tolerance question is given two points if the respondent reports needing more than two drinks; affirmative replies to the A, C, and E questions are each given one point. All the women agreed to receive structured brief advice and referral for a 15- to 20-minute motivational interviewing session with an alcohol health worker. The brief intervention group was compared with 150 women not treated with brief intervention (mean age 35+/-4). We also evaluated the impact of the support partner’s participation in the treatment. The intervention was offered during the first trimester. Statistical analysis was conducted using the Fisher exact test.

## Results

Brief intervention = abstinent 60.6% (151/251), consumption or reduction (C-R): 39.4% (100/251); no intervention = abstinent: 20% (30/150) p<0.001, C-R: 80% (120/150) p<0.001; Partner participation = abstinent 55.7% (140/251), C-R 44.3% (111/251) p<0.01.

## Conclusions

This experience evidences how brief intervention conducted by nonmedical professionals has significant implications for national public health policies. In cases where the partner participated, the effect of the intervention was significantly enhanced.

